# Supplementary material for: Iterative improvement in the automatic modular design of robot swarms
Source: PeerJ Comput Sci. 2020 Dec 7;6:e322. doi: 10.7717/peerj-cs.322 (PMC7924708; doi:10.7717/peerj-cs.322)
Supplement: Supplemental Information 3 [file peerj-cs-06-322-s003.zip › argos3/doc/api/standalone/a00583_source.html]

ARGoS: plugins/simulator/entities/perspective\_camera\_equipped\_entity.h Source File


- Main Page
- Related Pages
- Namespaces
- Classes
- Files

- File List
- File Members

# plugins/simulator/entities/perspective\_camera\_equipped\_entity.h

Go to the documentation of this file.

```
00001 
00007 #ifndef PERSPECTIVE_CAMERA_EQUIPPED_ENTITY_H
00008 #define PERSPECTIVE_CAMERA_EQUIPPED_ENTITY_H
00009 
00010 namespace argos {
00011    class CPerspectiveCameraEquippedEntity;
00012    class CEmbodiedEntity;
00013    struct SAnchor;
00014 }
00015 
00016 #include <argos3/core/simulator/entity/entity.h>
00017 #include <argos3/core/simulator/physics_engine/physics_model.h>
00018 #include <argos3/core/utility/math/vector3.h>
00019 #include <argos3/core/utility/math/quaternion.h>
00020 
00021 namespace argos {
00022 
00023    class CPerspectiveCameraEquippedEntity : public CEntity {
00024 
00025    public:
00026 
00027       ENABLE_VTABLE();
00028 
00029    public:
00030 
00036       CPerspectiveCameraEquippedEntity(CComposableEntity* pc_parent);
00037 
00052       CPerspectiveCameraEquippedEntity(CComposableEntity* pc_parent,
00053                                        const std::string& str_id,
00054                                        const CRadians& c_aperture,
00055                                        Real f_focal_length,
00056                                        Real f_range,
00057                                        SInt32 n_width,
00058                                        SInt32 n_height,
00059                                        SAnchor& s_anchor);
00060 
00065       virtual void Init(TConfigurationNode& t_tree);
00066 
00067       virtual void Enable();
00068 
00069       virtual void Disable();
00070 
00075       inline const CRadians& GetAperture() const {
00076          return m_cAperture;
00077       }
00078 
00083       inline void SetAperture(const CRadians& c_aperture) {
00084          m_cAperture = c_aperture;
00085       }
00086 
00091       inline Real GetFocalLength() const {
00092          return m_fFocalLength;
00093       }
00094 
00099       inline void SetFocalLength(Real f_focal_length) {
00100          m_fFocalLength = f_focal_length;
00101       }
00102 
00107       inline Real GetRange() const {
00108          return m_fRange;
00109       }
00110 
00115       inline void SetRange(Real f_range) {
00116          m_fRange = f_range;
00117       }
00118 
00123       inline const SAnchor& GetAnchor() const {
00124          return *m_psAnchor;
00125       }
00126 
00131       inline void SetAnchor(SAnchor& s_anchor) {
00132          m_psAnchor = &s_anchor;
00133       }
00134 
00139       inline SInt32 GetImagePxWidth() const {
00140          return m_nImagePxWidth;
00141       }
00142 
00147       inline SInt32 GetImagePxHeight() const {
00148          return m_nImagePxHeight;
00149       }
00150 
00156       inline void SetImagePxSize(SInt32 n_width,
00157                                  SInt32 n_height) {
00158          m_nImagePxWidth  = n_width;
00159          m_nImagePxHeight = n_height;
00160          Real fAspectRatio =
00161             static_cast<Real>(m_nImagePxWidth) /
00162             static_cast<Real>(m_nImagePxHeight);
00163          m_fImageMtHeight =
00164             2.0 * m_fFocalLength * Tan(m_cAperture) /
00165             Sqrt(1.0 + fAspectRatio * fAspectRatio);
00166          m_fImageMtWidth = m_fImageMtHeight * fAspectRatio;
00167       }
00168 
00173       inline Real GetImageMtWidth() const {
00174          return m_fImageMtWidth;
00175       }
00176 
00181       inline Real GetImageMtHeight() const {
00182          return m_fImageMtHeight;
00183       }
00184 
00185       virtual std::string GetTypeDescription() const {
00186          return "perspective_camera";
00187       }
00188 
00189    private:
00190 
00192       CRadians m_cAperture;
00193 
00195       Real m_fFocalLength;
00196 
00198       Real m_fRange;
00199 
00201       SInt32 m_nImagePxWidth;
00202 
00204       SInt32 m_nImagePxHeight;
00205 
00207       Real m_fImageMtWidth;
00208 
00210       Real m_fImageMtHeight;
00211 
00213       SAnchor* m_psAnchor;
00214 
00215    };
00216 }
00217 
00218 #endif
```

---

Generated on 10 Jul 2018 for ARGoS by 
 1.6.1
